# Complete Chloroplast Genome Features and Phylogenetic Analysis of *Linum usitatissimum* L.

**DOI:** 10.3390/genes16091038

**Published:** 2025-08-31

**Authors:** Qingqing Ji, Guanghui Du, Xingcai An, Junyuan Dong, Xiahong Luo, Changli Chen, Tingting Liu, Lina Zou, Shaocui Li, Jikang Chen, Xia An

**Affiliations:** 1 School of Agriculture, Yunnan University, Kunming 650500, China; jiqingqing1001@163.com (Q.J.); dgh2012@ynu.edu.cn (G.D.); xcan2001str@163.com (X.A.); 13964552682@163.com (J.D.); 2 Zhejiang Xiaoshan Institute of Cotton & Bast Fiber Crops, Zhejiang Institute of Landscape Plants and Flowers, Zhejiang Academy of Agricultural Sciences, Hangzhou 311251, China; luoxh@zaas.ac.cn (X.L.); chenchangli@zaas.ac.cn (C.C.); liutt@zaas.ac.cn (T.L.); zoulina1991@yeah.net (L.Z.); lishaocui@zaas.ac.cn (S.L.); 3 Institute of Bast Fiber Crops, Chinese Academy of Agricultural Sciences/Key Laboratory of Bast Fiber Biology and Processing, Ministry of Agriculture and Rural Affairs, Changsha 410221, China

**Keywords:** *Linum usitatissimum* L., chloroplast genome, phylogenetic analysis, genomic characterization

## Abstract

**Background:** The chloroplast genome provides rich genetic information for plant evolutionary studies. This study aimed to assemble, annotate, and analyze the complete chloroplast genome of flax cultivar ‘Longya 15’ (*Linum usitatissimum* L.) and clarify its phylogenetic relationships with other Linaceae species. **Methods:** We assembled and annotated the chloroplast genome of ‘Longya 15’ and retrieved chloroplast genomes of related species (e.g., *Linum grandiflorum* NC_058845.1, *Linum lewisii* NC_058799.1) from the NCBI database for phylogenetic analysis. **Results:** The chloroplast genome of ‘Longya 15’ was a 157,074-bp quadripartite structure with 37.42% GC content, encoding 128 genes (83 mRNAs, 37 tRNAs, 8 rRNAs) without pseudogenes. It showed codon bias for leucine (28 codons with RSCU > 1, ending in A/U), 260 dispersed repeats, and 240 SSRs. Ka/Ks analysis revealed purifying selection for most genes, while *rps18* and *ycf2* had positive selection. *ycf1* was identified as the hypervariable region (pi = 0.25024). Phylogenetically, it clustered closest with *Linum grandiflorum*, followed by *L. lewisii* and *L. perenne*, and was related to *Hypericum* species. **Conclusions:** This is the first fine assembly and annotation of ‘Longya 15’ chloroplast genome, confirming no pseudogenes in flax chloroplast. It elucidates flax chloroplast genome conservation and evolutionary dynamics, enriches the database, and provides a foundation for Linaceae phylogenetics, germplasm development, and stress-resistant breeding.

## 1. Introduction

Flax (*Linum usitatissimum* L.), an annual herbaceous plant of the genus *Linum* (family Linaceae), serves as both a bast fiber crop and oilseed crop. Based on economic utility, cultivated flax is classified into fiber flax, oil flax (linseed), and dual-purpose flax [1]. Globally, fiber flax is grown in over 20 countries while oil flax is cultivated in more than 40 regions, with China initiating flax cultivation in 1936 [2]. Notably, oil flax (locally termed “huma”) represents one of China’s five major oil crops, holding significant economic importance in arid northwest and north China [3]. Flaxseed has been traditionally used in medicine for centuries, containing abundant proteins, lipids, dietary fiber, α-linolenic acid (a key unsaturated fatty acid), and lignans that benefit human health. Studies confirm flaxseed’s efficacy in preventing cardiovascular diseases, diabetes, and obesity [4]. As a primary bast fiber source [5], flax exhibit exceptional tensile strength, softness, moisture absorption, antibacterial properties, and mildew resistance. Wearing linen fabrics in summer makes people feel cool and comfortable without a sticky or stuffy sensation; thus, linen fibers are praised as “breathable fibers” and “the queen of fibers”. The crop demonstrates strong adaptability, high biomass yield, and heavy metal tolerance [6]. In addition to medicinal and apparel applications, flax straw can be used to produce thermal insulation composites, biogas, furfural, and other products; meanwhile, flax shives and flax fibers can also be applied in papermaking. In recent years, research and application of flax fiber-reinforced composites have developed vigorously. The reinforcing effect of flax fibers in polymer matrices can improve the tensile properties, flexural properties, impact resistance, hardness, and other performance metrics of the matrices, with synergistic effects achieved through the combination of multiple types of fibers. Compared with corresponding petroleum-based fiber composites, flax fiber composites are more environmentally friendly. Currently, flax fiber composites have been able to replace plastics and are applicable in automotive interiors as well as front and rear casings [7].Recently, flax fibers have gained extensive applications in textiles, industrial manufacturing, healthcare, food additives, advanced materials, and papermaking, highlighting their substantial economic value [8,9].

Flax cultivar ‘Longya 15’ was selected for this study, with the core basis derived from three aspects of research gaps and industrial demands: first, flax is a dual-purpose crop for oil and fiber. Existing chloroplast studies mostly focus on common cultivars [10]; however, as a major cultivar in the arid region of Northwest China, the chloroplast genome structure and functional gene characteristics of ‘Longya 15’ have not been clarified, which fails to support targeted breeding. Second, there is controversy regarding the phylogenetic position of flax in Linaceae—conclusions from Fu et al. [11] contradict with those from Lopes et al. [10]—and chloroplast data of this cultivar is lacking verification. Third, stress conditions cause a yield reduction of over 30% in flax [3]; yet, only a few chloroplast stress-resistant genes have clear functions, while the roles of most genes (e.g., the *ycf* family) remain to be elucidated, requiring genome-level research to fill this gap.

The plant chloroplast is a semi-autonomous genetic organelle with a double-membrane structure [12] that directly determines crop yield by converting light energy into ATP and carbohydrate energy. When adverse environmental factors such as drought, flooding, salinity, extreme temperatures, nutrient imbalance, pathogens, and viruses interfere with photosynthetic function, they can cause significant yield reduction. Chloroplasts possess two membrane structures: the double envelope (inner and outer membranes) and the thylakoid membrane, each equipped with specific ion channels and transport proteins that efficiently mediate the transmembrane transport of nutrients, solutes, and metabolites [13]. In recent years, with the rapid development of high-throughput sequencing technologies, significant progress has been made in the study of plant chloroplast genomes. The chloroplast DNA typically exists as a double-stranded circular molecule, with most higher plants exhibiting a highly conserved quadripartite structure in their chloroplast genomes [14]. This structure consists of two inverted repeat regions (IR) separated by a large single-copy region (LSC) and a small single-copy region (SSC), typically ranging from 107 to 218 kb in length. Characterized by highly conserved genomes, slow evolutionary rates, and maternal uniparental inheritance [15], chloroplasts play significant roles in plant phylogenetic studies, elucidation of photosynthetic molecular mechanisms, and genetic engineering [16].

*Malpighiales*, one of the largest orders of angiosperms, exhibits remarkable morphological and ecological diversity, holding significant ecological and economic importance [17]. The family Linaceae occupies a crucial position within Malpighiales, comprising approximately 200 species. As a core member of Linaceae, flax (*L. usitatissimum*) represents the most economically valuable species in the genus. However, the phylogenetic position of flax within Linaceae and its evolutionary relationships with closely related species remain unclear. Chloroplast genomes provide valuable genetic information for plant evolutionary studies. In this study, we selected representative flax varieties and employed high-throughput sequencing technologies combined with bioinformatics approaches to sequence, assemble, and annotate their chloroplast genomes, followed by comprehensive analysis of structural characteristics and functional genes. Furthermore, phylogenetic analysis was conducted to elucidate flax’s evolutionary position within Linaceae and *Malpighiales*. These findings will provide important theoretical foundations for phylogenetic studies of Linaceae plants and the conservation and utilization of germplasm resources.

This study intends to characterize the chloroplast genome of flax cultivar ‘Longya 15’, and subsequent research can be advanced focusing on key knowledge gaps as follows: first, if hypervariable regions are identified, molecular markers can be developed to improve the efficiency of flax germplasm differentiation, thereby facilitating the screening of elite cultivars. Second, combined with stress experiments, the functions of candidate chloroplast stress-resistant genes (e.g., the *ndh* and *ycf* families) can be explored to provide targets for stress-resistant breeding. Third, by integrating multi-omics data, the phylogenetic relationships within Linaceae can be clarified, and the taxonomic framework of *Malpighiales* can be improved, laying a foundation for similar studies.

## 2. Materials and Methods

### 2.1. Plant Materials and Sequencing

The experimental material was flax cultivar ‘Longya 15’ (*L. usitatissimum*), cultivated at the Zhejiang Institute of Landscape Plants and Flowers (Zhejiang Xiaoshan Cotton and Bast Fiber Crops Research Institute, Xiaoshan, China) (30°07′ N, 120°23′ E). Healthy young leaves were collected, cleaned to remove impurities, dried thoroughly to eliminate residual moisture, and immediately flash-frozen in liquid nitrogen for 10 min in pre-chilled EP tubes before storage at −80 °C. Total genomic DNA was extracted using a universal plant DNA extraction kit (GeneBetter D312), followed by paired-end (PE) sequencing on the Illumina NovaSeq 6000 platform.

The raw sequencing data of flax ‘Longya 15’ (including paired-end reads) and its annotated chloroplast genome (FASTA format + GFF3 annotation) have been submitted to the NCBI GenBank database. The accession number is pending official assignment (Submission ID: 2997594); sequencing platform: Illumina NovaSeq 6000; assembly pipeline: GetOrganelle v1.7.7.1 + manual correction; annotation tools: Prodigal v2.6.3, HMMER v3.1b2, Aragorn v1.2.38).

### 2.2. Chloroplast Genome Assembly and Functional Annotation

The raw sequencing data were quality-filtered using fastp v0.23.4 [18] to remove adapter/primer sequences and discard reads with average quality scores < Q5 or containing >5 ambiguous bases (N), generating clean data for subsequent analysis. Chloroplast genome assembly was performed using GetOrganelle v1.7.7.1 with default parameters. For comprehensive genome annotation, we implemented a dual-approach strategy: (i) de novo prediction using Prodigal v2.6.3 [19] for protein-coding genes (CDS), HMMER v3.1b2 [20] for rRNA identification, and Aragorn v1.2.38 [21] for tRNA detection; and (ii) homology-based annotation through BLAST v2.6 [22] alignment against published chloroplast genomes of closely related species from NCBI. The two annotation results were systematically integrated through manual curation to resolve discrepancies, including removal of erroneous/redundant annotations and precise determination of exon–intron boundaries for multi-exon genes. The final annotated chloroplast genome was visualized using OGDRAW [23] to generate the complete genomic map.

### 2.3. Analysis of Dispersed and Simple Sequence Repeats

Dispersed repeats were identified using vmatch v2.3.0 [24] with customized Perl scripts, with parameter settings including the following: minimum length = 30 bp, Hamming distance = 3, and four detection modes (forward, palindromic, reverse, and complement). For chloroplast simple sequence repeat (cpSSR) analysis, MISA v1.0 [25] was employed with the following thresholds: mononucleotide repeats ≥8 units, dinucleotide repeats ≥5 units, and tri-, tetra-, penta-, and hexanucleotide repeats ≥3 units each.

### 2.4. Analysis of Chloroplast Genome Nucleotide Diversity and Boundary Regions

The chloroplast genomes of eight species from Linaceae and Hypericaceae were downloaded from NCBI, including *Linum grandiflorum* (NC_058845.1), *L. lewisii* (NC_058799.1), *Linum narbonense* (NC_058855.1), *Hypericum perforatum* (NC_083133.1), *Hypericum ascyron* (MZ424306.1), *Hypericum monogynum* (NC_069025.1), and *Hypericum sampsonii* (PQ638954.1); the eighth was the chloroplast genome of flax ‘Longya 15’ (*L. usitatissimum*) assembled in this study. Among these, the chloroplast genome of flax ‘Longya 15’ (*L. usitatissimum*) was newly sequenced and annotated in this study (Submission ID: 2997594), while other genomes were retrieved from public repositories. Global alignment of homologous gene sequences was performed using MAFFT (v7.427—auto mode), followed by calculation of pi values for each gene using DnaSP5 v5.10.1 [26]. Boundary regions were visualized using CPJSdraw (http://cloud.genepioneer.com:9929/#/tool/alltool/detail/296, accessed on 1 July 2025) from GenePioneer’s cloud platform, and whole genome alignments were conducted with Mauve (v2.3.1) [27] using default parameters.

### 2.5. Methods for Phylogenetic Analysis

The chloroplast genome sequences of 19 *Malpighiales* species were retrieved from the NCBI database, where the 20th was the genome of flax ‘Longya 15’ (this study). *Prunus persica* (Rosaceae) was selected as the outgroup for phylogenetic reconstruction. Among these, the chloroplast genome of flax ‘Longya 15’ (*L. usitatissimum*) was newly sequenced and annotated in this study (Submission ID: 2997594), while other genomes were retrieved from public repositories. Shared CDS sequences were analyzed through the following pipeline: (1) multiple sequence alignment performed using MAFFT v7.427 (—auto mode); (2) unreliable alignment regions removed and CDS sequences concatenated using trimAl v1.4.rev15 [28]; (3) optimal nucleotide substitution model selected under Bayesian Information Criterion using jModelTest v2.1.10; and (4) maximum likelihood phylogenetic tree constructed with RAxML v8.2.10 [29] employing the GTRGAMMA model and 1000 rapid bootstrap replicates.

## 3. Results

### 3.1. General Characteristics of the Flax Chloroplast Genome

**Figure 1:**
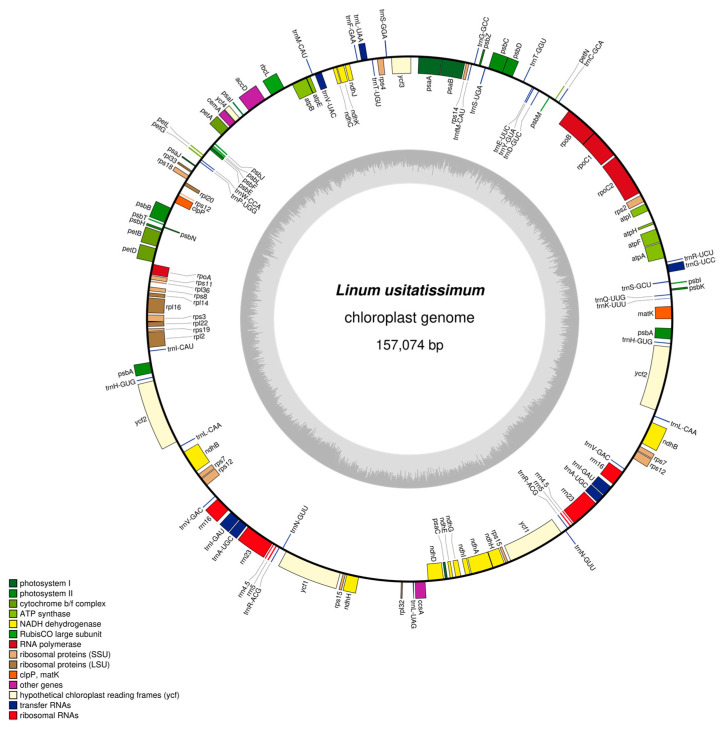
Map of *Linum usitatissimum* L. chloroplast genome. Note: genes encoded on the forward strand are displayed on the outer circle, while those encoded on the reverse strand are shown on the inner circle. The gray inner ring represents GC content distribution.

The flax chloroplast genome exhibits a typical quadripartite structure with a total length of 157,074 bp, comprising two inverted repeat regions (IRa and IRb, each 32,166 bp), a large single-copy region (LSC, 81,769 bp), and a small single-copy region (SSC, 10,973 bp) (Figure 1, Table 1). Nucleotide composition analysis revealed the following base distribution: A (30.94%), C (18.97%), G (18.44%), and T (31.64%). The overall GC content of the chloroplast genome was 37.42%, with IR regions showing higher GC content (40.31% for both IRa and IRb) compared to LSC (35.89%) and SSC (31.91%) regions (Table 1).

### 3.2. Functional Annotation of Flax Chloroplast Genes

The *L. usitatissimum* chloroplast genome was annotated with a total of 128 genes, comprising 83 mRNA genes, 37 tRNA genes, and 8 rRNA genes, with no pseudogenes detected (Table 2). These genes primarily function in photosynthesis and self-replication processes, along with additional genes involved in auxiliary chloroplast metabolism, protein processing, and membrane structure maintenance, while the functions of some genes remain uncharacterized. Gene copy number analysis revealed 57 mRNAs and 21 tRNAs presented as single copies, while 11 mRNAs, 14 tRNAs, and 4 rRNAs existed as double copies. Intron analysis showed 10 mRNAs and 8 tRNAs containing one intron, and 2 mRNAs possessing two introns (Table 2).

To validate the accuracy of gene annotation, we compared the annotated genes of ‘Longya 15’ with those of *Linum grandiflorum* (NC_58845.1, retrieved from NCBI). The results showed high conservation: ‘Longya 15’ has 128 genes (83 mRNAs, 37 tRNAs, 8 rRNAs), while *Linum grandiflorum* has 129 genes (84 mRNAs, 37 tRNAs, 8 rRNAs). The slight difference (1 mRNA) may be due to cultivar-specific variation, which is consistent with the structural conservation of chloroplast genomes in Linaceae.

### 3.3. Codon Usage Bias Analysis

**Figure 2:**
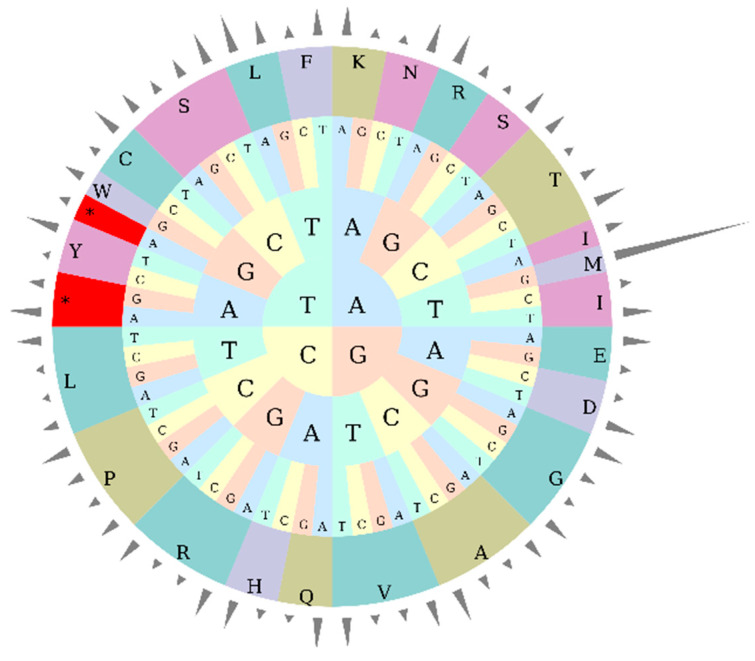
Circular visualization of relative synonymous codon usage (RSCU) in the flax chloroplast genome. Notes: Different colors represent distinct amino acids (abbreviated on the outer ring, e.g., L for Leucine, F for Phenylalanine, etc.); the inner letters (A, T, C, G) denote nucleotides; asterisks (*) indicate codons with notable characteristics.

Systematic analysis of codon usage patterns in the *L. usitatissimum* chloroplast genome revealed 22,661 codons participating in amino acid encoding (excluding termination codons *Ter*). Among all amino acid-specific codons and leucine (Leu) codons showed the highest usage frequency (2384 occurrences), followed by serine (Ser; 1613) and isoleucine (Ile; 1820). Relative synonymous codon usage (RSCU) analysis demonstrated that 28 codons exhibited RSCU > 1, with 26 terminating in A/U, while 35 codons showed RSCU < 1, including 32 G/C-ending codons. Notably, tryptophan (Trp) was exclusively encoded by UGG (RSCU = 1). The start codon AUG (methionine, Met) displayed the highest RSCU value (6.986), followed by UUA (Leu; 1.986) and GCU (alanine, Ala; 1.6904), whereas GUG (Met) showed the lowest RSCU (0.014) (Table 3). The circular codon composition diagram (Figure 2) visually represents the distribution patterns of amino acid-specific codons, providing critical insights for deciphering codon usage preferences in the flax chloroplast genome.

### 3.4. Repeat Sequence Analysis

**Figure 3:**
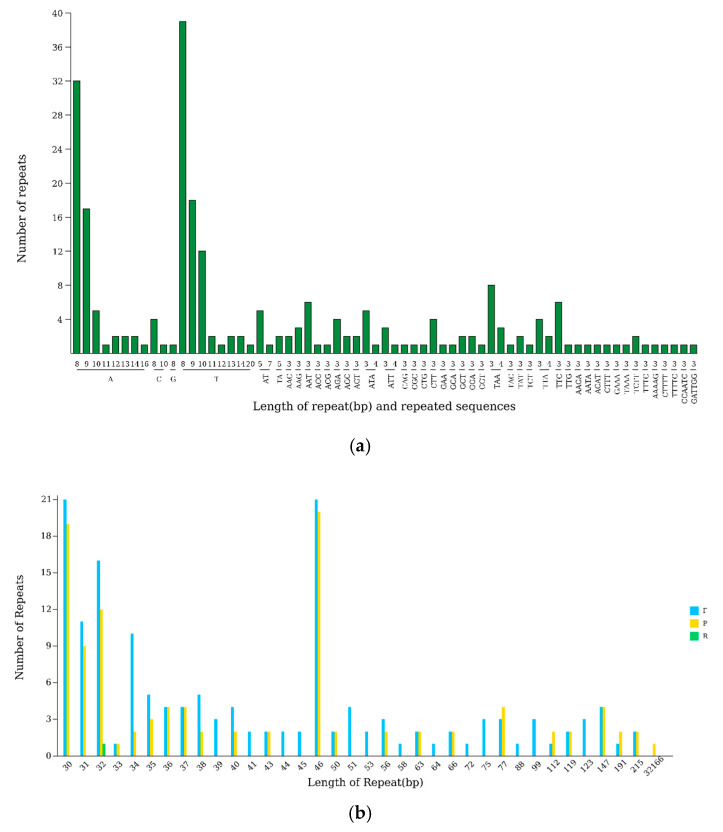
(**a**) Scattered and simple sequence repeats in *L. usitatissimum* L. chloroplast genome. (**b**) Length distribution of repeats in *L. usitatissimum* L. chloroplast genome.

The *L. usitatissimum* chloroplast genome contains 260 dispersed repeats, comprising 154 forward (F), 105 palindromic (P), 1 reverse (R), and 0 complementary (C) repeats. Size distribution analysis revealed that most dispersed repeats (30–215 bp) were concentrated at 46 bp (41 repeats) and 30 bp (40 repeats), with one exceptionally long repeat spanning 32,166 bp (Figure 3a). Simple sequence repeats (SSRs), consisting of tandemly repeated 1–6 nucleotide motifs, were identified with 240 occurrences distributed across the chloroplast genome: 139 in the large single-copy (LSC) region, 23 in the small single-copy (SSC) region, and 78 in the inverted repeat (IR) regions. Genomic compartmentalization analysis showed that the LSC region contained 46 exonic, 14 intronic, and 79 intergenic SSRs; the SSC region had 4 exonic and 19 intergenic SSRs (with no intronic SSRs); while the IRs contained 53 exonic, 1 intronic, and 24 intergenic SSRs.

Mononucleotide SSRs exhibited the greatest diversity, with poly-A (8–16 repeats, 1–32 occurrences) and poly-T (8–20 repeats, 1–39 occurrences) being predominant. Dinucleotide repeats (e.g., AT/TA) and trinucleotide repeats (e.g., AAC/AAG) were also identified, along with fewer tetra-, penta-, and hexanucleotide repeats. Frequency analysis of all 240 SSRs revealed the top three prevalent motifs as T(8) (16.25%, 39 occurrences), A(8) (13.33%, 32 occurrences), and T(9) (7.5%, 18 occurrences) (Figure 3b).

### 3.5. Nucleotide Diversity and Boundary Analysis

**Figure 4:**
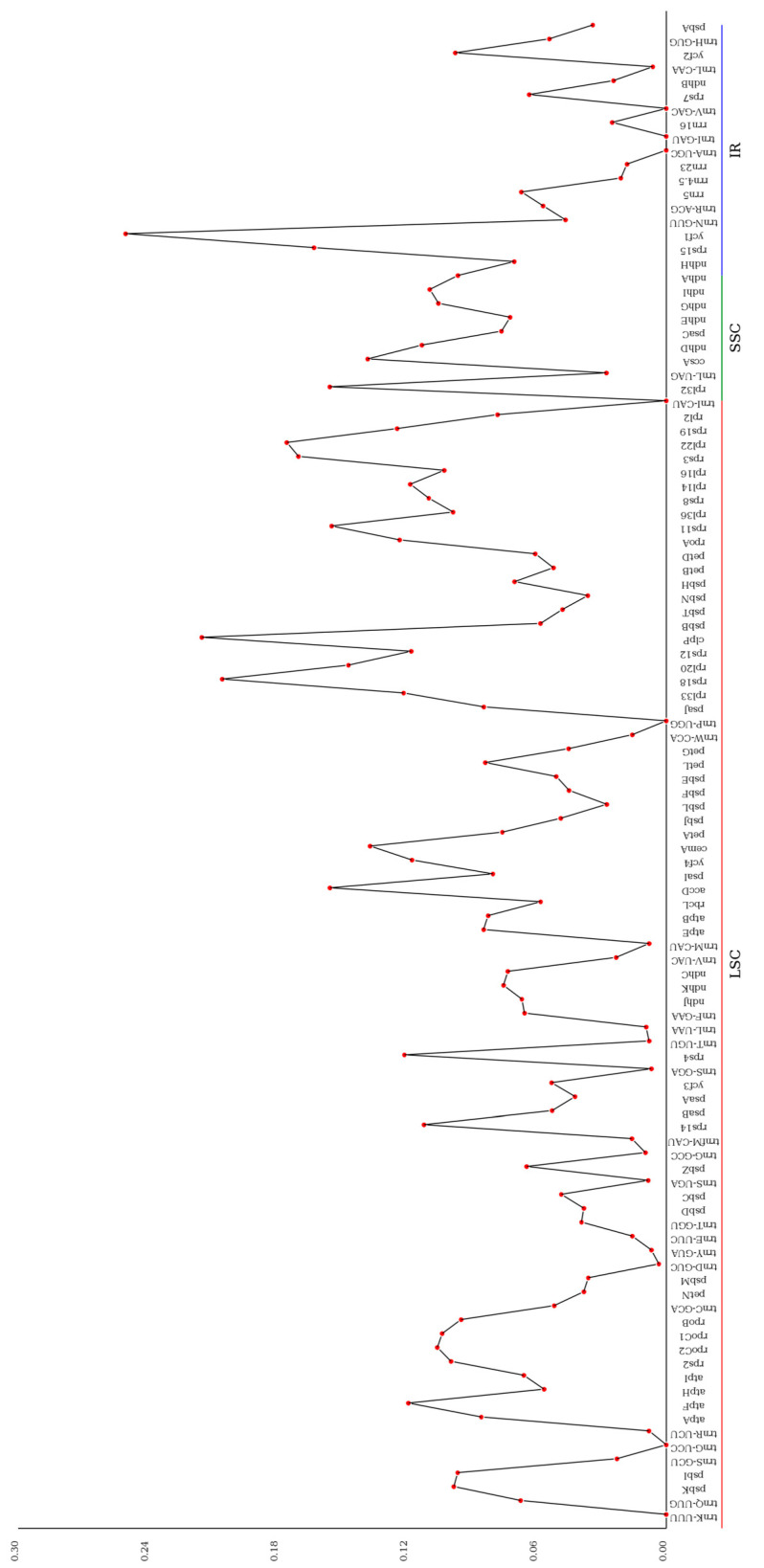
Line chart of gene Pi value.

Nucleotide diversity analysis of the *L. usitatissimum* chloroplast genome revealed an average pi value of 0.0735 across all 108 examined gene regions (Figure 4). Regional distribution analysis showed that the small single-copy (SSC) region exhibited the highest nucleotide diversity (pi = 0.1123), followed by the large single-copy (LSC) region (pi = 0.0786), while the inverted repeat (IR) regions displayed the lowest diversity (pi = 0.0521). Through screening, we identified 28 highly variable regions (pi ≥ 0.02), including 20 loci in LSC (e.g., *rps18* [0.20551], *clpP* [0.21494], *rpl33* [0.12154]), 6 loci in IR (e.g., *ycf1* [0.25024], *rps15* [0.16304], *ndhH* [0.07031]), and 2 loci in SSC (*ccsA* [0.13816], *rpl32* [0.1557]). The most variable locus was *ycf1* (pi = 0.25024) located in the IR region.

During the evolutionary process of plant chloroplast genomes, the expansion and contraction of IR (inverted repeat) boundaries represent key factors contributing to size variations. Comparative analysis of four Linaceae and four *Hypericaceae* species revealed four conserved boundary junctions in their chloroplast genomes: JLB (LSC/IRb), JSB (IRb/SSC), JSA (SSC/IRa), and JLA (IRa/LSC). Key genes adjacent to IR boundaries included *rpl2*, *ycf1*, and *trnH*. In *Linaceae*, the JLB boundary was located within the *rpl2* coding region, exhibiting minimal positional variation (1–2 bp) among species. The JSB boundary resided within *ycf1*, with the majority of this gene (all but 2–3 bp) positioned in IRb. The JSA boundary also occurred within *ycf1*, spanning 1027–1030 bp in IRa and 4271–4358 bp in SSC. The trnH gene was consistently located in LSC, 2–30 bp from the JLA boundary.

**Figure 5:**
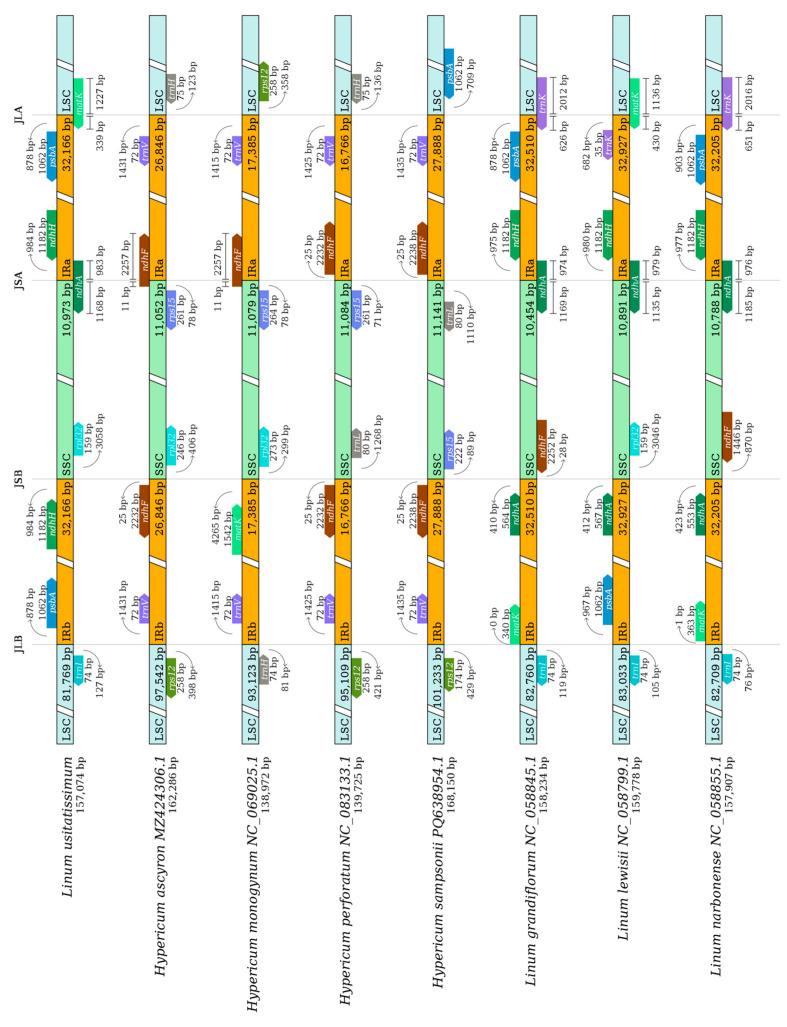
IR/SC boundary analysis.

*Hypericaceae* species showed similar patterns: the JLB boundary within *rpl2* (1–2 bp variation), the JSB boundary overlapping *ycf1* and *ndhF* (36–37 bp overlap), *ycf1* predominantly in IRb (2–3 bp in SSC), the JSA boundary within *ycf1* (1027–1030 bp in IRa; 4271–4358 bp in SSC), and *trnH* in LSC (2–30 bp from JLA) (Figure 5). These results demonstrate high structural conservation between *Linaceae* and *Hypericaceae* chloroplast genomes, with IR boundaries exhibiting limited variation affecting only a few genes.

### 3.6. Ka/Ks Analysis

**Figure 6:**
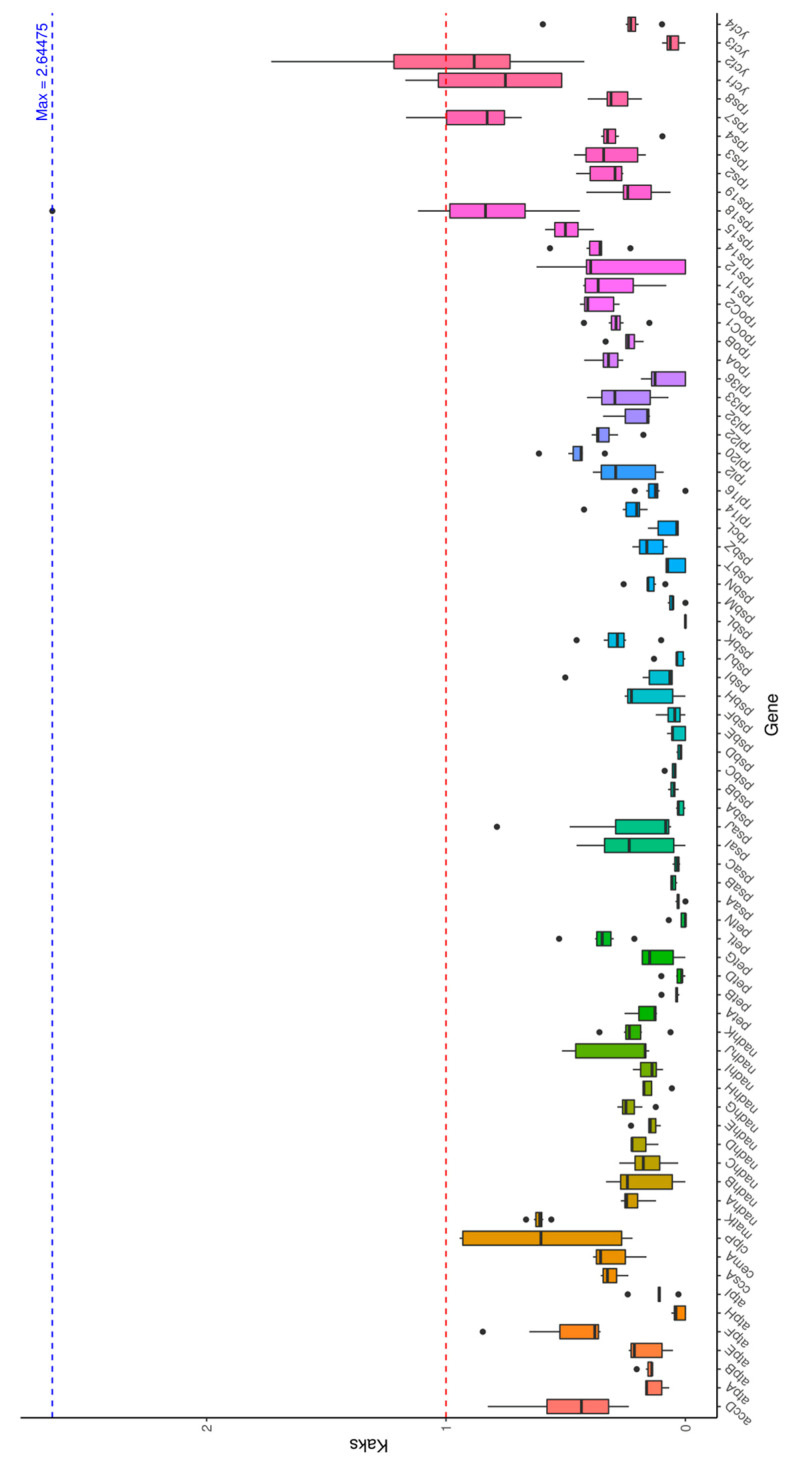
Ka/Ks analysis.

The Ka/Ks analysis of chloroplast genes between *L. usitatissimum* and seven related species (Figure 6) revealed an overall average ratio of 0.32, with most genes (e.g., *atpA*, *psaA*) exhibiting Ka/Ks < 1, indicating purifying selection and functional conservation. Notably, *atpH* showed Ka/Ks = 0 when compared with some *Linaceae* species, demonstrating exceptionally strong conservation. Among highly variable genes, *rps18* displayed the maximum Ka/Ks value (2.64) when compared with NC_058855, while *ycf2* showed a Ka/Ks ratio of 1.73 against the same reference, both >1 suggesting positive selection. Interspecific comparisons revealed slightly higher Ka/Ks values between flax and *Hypericaceae* species than within *Linaceae*, consistent with their phylogenetic divergence.

### 3.7. Phylogenetic Analysis

**Figure 7:**
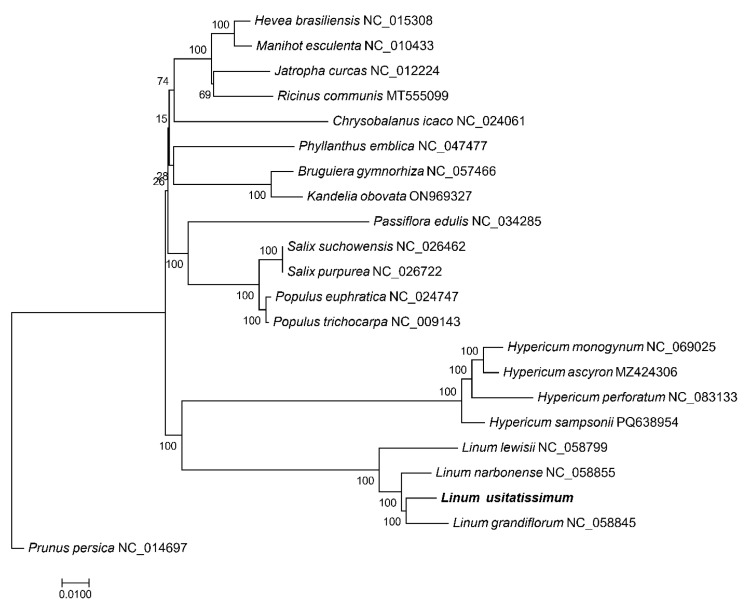
Phylogenetic tree constructed based on chloroplast genome sequences.

To comprehensively elucidate evolutionary relationships within *Malpighiales*, chloroplast genome sequences of 22 species were retrieved from NCBI, with Prunus persica (Rosaceae) designated as the outgroup. The phylogenetic reconstruction demonstrated that *L. usitatissimum* exhibited closest affinity with *Linum grandiflorum*, followed by congeneric species *L. lewisii* and *Linum narbonense*. Subsequent clustering revealed close evolutionary relationships with *Hypericum* species (*H. perforatum*, *H. ascyron*, *H. monogynum*, and *H. sampsonii*), indicating *Hypericum* as the phylogenetically nearest genus to *Linum* within *Malpighiales*. More distant relationships were observed with Salicaceae species (*Populus trichocarpa*, *P. euphratica*, *Salix suchowensis*, and *S. purpurea*), while Euphorbiaceae members (*Hevea brasiliensis*, *Manihot esculenta*, *Ricinus communis*, *Jatropha curcas*, and *Phyllanthus emblica*) showed the most substantial phylogenetic divergence. As expected, the outgroup *P. persica* occupied the most basal position (Figure 7).

## 4. Discussion

The chloroplast genome of *L. usitatissimum* assembled in this study measures 157,074 bp in length, displaying the characteristic quadripartite structure typical of most land plants. The observed GC content (37.42%) is marginally lower than previously reported values for flax plastomes (37.5%) [10], potentially reflecting cultivar-specific variations or methodological differences in sequencing and assembly. Our annotation identified 128 protein-coding genes with no pseudogenes detected, contrasting with the two pseudogenes (*rpl23* and *ndhF*) reported by Lopes et al. [10]—this discrepancy likely stems from differing pseudogene identification criteria and requires further functional validation.

Analysis of repetitive elements revealed a predominance of mononucleotide simple sequence repeats (SSRs) accounting for 72.70% of total repeats, consistent with the AT-rich SSR profile described by Lopes et al. [10] suggesting evolutionary conservation of these motifs within Linaceae that could serve as reliable molecular markers for population genetics. The *ycf1* gene exhibited the highest nucleotide diversity (pi = 0.25024), aligning with its established role as a hypervariable region across plant species, potentially related to its functional involvement in chloroplast protein translocation machinery. Comparative analysis with nuclear genome data from Melnikova et al. (2019) [30] revealed an inverse relationship between dispersed repeats (e.g., *Athila*-family *Ty3/gypsy* retrotransposons) and satellite DNA content in Linum species—cultivated flax (2n = 30) showed elevated satellite DNA (~13%) but reduced dispersed repeats. This contrasts markedly with our chloroplast genome findings of 260 dispersed repeats and 240 SSRs dominated by specific types (e.g., forward repeats, mononucleotide repeats), collectively demonstrating high structural conservation. These divergent patterns reflect distinct evolutionary strategies: nuclear genomes employ satellite DNA amplification for chromatin stability under environmental stress, while chloroplast genomes maintain conserved repeat architectures to preserve photosynthetic functionality.

Recent studies are highly consistent with the results of this study: Ji et al. [5], in their research on the salt tolerance mechanism of flax, found that variations in the hypervariable region of the *ycf1* gene were significantly correlated with salt stress tolerance, which confirms the value of *ycf1* (pi = 0.25024) as a stress-resistant candidate gene in this study. Dang et al. [3] pointed out that flax SSR markers can shorten the breeding cycle by 30%, and the 240 SSRs identified in this study (including 139 in the LSC region) provide direct targets for the development of such markers. In addition, Bolsheva et al. [30] reported that flax satellite DNA is associated with genome stability, which contrasts with the conservation of 260 dispersed repeats in the chloroplast genome observed in this study, suggesting that the nuclear–plastid genome co-evolution mechanism is worthy of further exploration.

Inverted repeat (IR) boundary analysis demonstrated substantial conservation between *Linaceae* and *Hypericaceae*, with only minor positional variations affecting few genes, resembling the IR stability observed in Brassicaceae but differing from the IR expansion/contraction events reported by Lopes et al. [10]—these discrepancies may reflect analytical or sampling differences. Ka/Ks analysis comparing flax with seven relatives showed predominant purifying selection (Ka/Ks < 1) across most genes, with exceptional conservation in *atpH* (Ka/Ks = 0), while *rps18* and *ycf2* exhibited positive selection (Ka/Ks > 1), potentially linked to environmental adaptation. The slightly elevated inter-family versus intra-family ratios support Lopes et al.’s [10] observation of accelerated evolutionary rates in these genomic regions.

Phylogenetic reconstruction clustered flax with congeneric species (*L. grandiflorum*, *L. lewisii*), then with *Hypericum* species, supporting close *Linaceae–Hypericaceae* relationships within *Malpighiales* contrasting with Lopes et al.’s [10] proposed closer affinity to *Chrysobalanaceae*, possibly reflecting methodological or outgroup selection differences. Fu et al. [11] confirmed two major *Linum* lineages (*Linum–Dasylinum* and *Linastrum–Syllinum* groups) through multi-genome SNP analysis, with cultivated flax showing closest relationships to *L. decumbens* and *L. grandiflorum*—results perfectly congruent with our whole-plastome phylogeny. The phylogenetic network approach employed by Fu et al. [31] provides complementary methodological insights to our tree-based analysis, suggesting future integration of network models for plastome evolution studies.

Collectively, these studies underscore the genetic value of wild relatives: Bolshev [30] identified chromosome-30-specific satellite DNA patterns, while Fu [11,31] highlighted wild species as reservoirs of stress-tolerance genes. Lopes et al.’s [10] foundational flax plastome research revealed atypical structural evolution, novel RNA editing sites, and proposed *Linaceae*’s phylogenetic position near *Chrysobalanaceae*. Our findings demonstrate chloroplast genomes’ unique utility for intragenus phylogenetics, with noncoding regions better suited for intraspecific differentiation, while whole-plastome data excel in interfamily comparisons. Future studies should integrate multi-genome approaches with network modeling to resolve *Linaceae–Hypericaceae* relationships and clarify *Linaceae*’s position relative to *Chrysobalanaceae* within *Malpighiales*.

To ensure the reproducibility of this study, all original data (including sequencing reads and annotated genomes) have been submitted to NCBI GenBank (Submission ID: 2997594), enabling other researchers to verify our phylogenetic and comparative analyses.

## 5. Conclusions

This study elucidates fundamental characteristics of the *L. usitatissimum* chloroplast genome: a 157,074 bp circular DNA with typical quadripartite structure, 37.42% GC content, 128 annotated genes, and codon usage bias favoring A/U-ending codons, dominated by mononucleotide repeats. Nucleotide diversity analysis identified the small single-copy (SSC) region as the most variable (pi = 0.1123) and inverted repeats (IRs) as the most conserved (pi = 0.0521), with 28 hypervariable regions (e.g., *ycf1*, *clpP*) serving as potential molecular markers for Linaceae species identification and phylogenetic studies. Comparative IR boundary analysis revealed high structural conservation between *Linaceae* and *Hypericaceae* with only minor variations. Phylogenetic reconstruction demonstrated closest relationships among Linum species, with *Hypericum* as the nearest relative, providing molecular evidence for *Linaceae* taxonomy and evolution.

These findings provide baseline data for flax chloroplast genomics, where identified hypervariable regions and repetitive elements offer practical tools for germplasm characterization and genetic improvement. The phylogenetic results provide novel insights into evolutionary relationships within *Malpighiales*, establishing a framework for future comparative genomic studies in this economically important plant order.

## Figures and Tables

**Table 1:** Chloroplast genome characteristics of *Linum usitatissimum* L.

Region	A Content/%	C Content/%	G Content/%	T Content/%	GC Content/%	Base Length/bp
LSC	31.03	18.45	17.43	33.08	35.89	81,769
SSC	36.71	15.97	15.94	31.39	31.91	10,973
IRa	28.89	18.58	21.72	30.80	40.31	32,166
IRb	30.80	18.58	21.72	28.89	40.31	32,166
Total	30.94	18.97	18.44	31.64	37.42	157,074

**Table 2:** Gene annotation of the chloroplast genome of *L. usitatissimum*.

Category	Gene Group	Gene Name
Photosynthesis	Subunits of photosystem I	*psaA*, *psaB*, *psaC*, *psaI*, *psaJ*
	Subunits of photosystem II	*psbA(2)*, *psbB*, *psbC*, *psbD*, *psbE*, *psbF*, *psbH*, *psbI*, *psbJ*, *psbK*, *psbL*, *psbM*, *psbN*, *psbT*, *psbZ*
	Subunits of NADH dehydrogenase	ndhA *, ndhB *(2), *ndhC*, *ndhD*, *ndhE*, *ndhG*, *ndhH(2)*, *ndhI*, *ndhJ*, *ndhK*
	Subunits of cytochrome b/f complex	*petA*, petB *, petD *, *petG*, *petL*, *petN*
	Subunits of ATP synthase	*atpA*, *atpB*, *atpE*, atpF *, *atpH*, *atpI*
	Large subunit of rubisco	*rbcL*
	Subunits photochlorophyllide reductase	-
Self-replication	Proteins of large ribosomal subunit	*rpl14*, rpl16 *, rpl2 *, *rpl20*, *rpl22*, *rpl32*, *rpl33*, *rpl36*
	Proteins of small ribosomal subunit	*rps11*, rps12 **(2), *rps14*, *rps15(2)*, *rps18*, *rps19*, *rps2*, *rps3*, *rps4*, *rps7(2)*, *rps8*
	Subunits of RNA polymerase	*rpoA*, *rpoB*, rpoC1 *, *rpoC2*
	Ribosomal RNAs	*rrn16(2)*, *rrn23(2)*, *rrn4.5(2)*, *rrn5(2)*
	Transfer RNAs	trnA-UGC *(2), *trnC-GCA*, *trnD-GUC*, *trnE-UUC*, *trnF-GAA*, *trnG-GCC*, trnG-UCC *, *trnH-GUG(2)*, *trnI-CAU*, trnI-GAU *(2), trnK-UUU *, *trnL-CAA(2)*, trnL-UAA *, *trnL-UAG*, *trnM-CAU*, *trnN-GUU(2)*, *trnP-UGG*, *trnQ-UUG*, *trnR-ACG(2)*, *trnR-UCU*, *trnS-GCU*, *trnS-GGA*, *trnS-UGA*, *trnT-GGU*, *trnT-UGU*, *trnV-GAC(2)*, trnV-UAC *, *trnW-CCA*, *trnY-GUA*, *trnfM-CAU*
Other genes	Ribosomal RNAs	*rrn16(2)*, *rrn23(2)*, *rrn4.5(2)*, *rrn5(2)*
	Transfer RNAs	trnA-UGC *(2), *trnC-GCA*, *trnD-GUC*, *trnE-UUC*, *trnF-GAA*, *trnG-GCC*, trnG-UCC *, *trnH-GUG(2)*, *trnI-CAU*, trnI-GAU *(2), trnK-UUU *, *trnL-CAA(2)*, trnL-UAA *, *trnL-UAG*, *trnM-CAU*, *trnN-GUU(2)*, *trnP-UGG*, *trnQ-UUG*, *trnR-ACG(2)*, *trnR-UCU*, *trnS-GCU*, *trnS-GGA*, *trnS-UGA*, *trnT-GGU*, *trnT-UGU*, *trnV-GAC(2)*, trnV-UAC *, *trnW-CCA*, *trnY-GUA*, *trnfM-CAU*
	Maturase	matK *
	Protease	*clpP*
	Envelope membrane protein	*cemA*
	Acetyl-CoA carboxylase	*accD*
	c-type cytochrome synthesis gene	*ccsA*
	Translation initiation factor	-
	Other	-
Genes of unknown function	Conserved hypothetical chloroplast ORF	*ycf1(2)*, *ycf2(2)*, ycf3 **, *ycf4*

Note: gene * indicates genes containing one intron; gene ** denotes genes with two introns; gene (2) signifies genes with copy numbers > 1 (the specific copy number is shown in parentheses).

**Table 3:** Relative synonymous codon usage analysis of *L. usitatissimum*.

Amino Acid	Codon	Count	RSCU	Amino Acid	Codon	Count	RSCU	Amino Acid	Codon	Count	RSCU
Ter	UAA	42	1.68	Ile	AUA	543	0.8952	Arg	AGA	332	1.56
Ter	UAG	20	0.8001	Ile	AUC	333	0.549	Arg	AGG	146	0.6858
Ter	UGA	13	0.5199	Ile	AUU	944	1.5561	Arg	CGA	307	1.4424
Ala	GCA	327	1.0388	Lys	AAA	957	1.5048	Arg	CGC	113	0.531
Ala	GCC	231	0.734	Lys	AAG	315	0.4952	Arg	CGG	111	0.5214
Ala	GCG	169	0.5368	Leu	CUA	322	0.8106	Arg	CGU	268	1.2594
Ala	GCU	532	1.6904	Leu	CUC	165	0.4152	Ser	AGC	117	0.435
Cys	UGC	68	0.5552	Leu	CUG	137	0.345	Ser	AGU	324	1.2054
Cys	UGU	177	1.4448	Leu	CUU	491	1.236	Ser	UCA	292	1.086
Asp	GAC	196	0.438	Leu	UUA	789	1.986	Ser	UCC	254	0.945
Asp	GAU	699	1.562	Leu	UUG	480	1.2078	Ser	UCG	185	0.6882
Glu	GAA	904	1.4688	Met	AUG	502	6.986	Ser	UCU	441	1.6404
Glu	GAG	327	0.5312	Met	GUG	1	0.014	Thr	ACA	294	1.1104
Phe	UUC	380	0.613	Asn	AAC	241	0.4662	Thr	ACC	209	0.7896
Phe	UUU	860	1.387	Asn	AAU	793	1.5338	Thr	ACG	122	0.4608
Gly	GGA	577	1.5028	Pro	CCA	234	1.0344	Thr	ACU	434	1.6392
Gly	GGC	185	0.4816	Pro	CCC	202	0.8928	Val	GUA	414	1.38
Gly	GGG	288	0.75	Pro	CCG	133	0.588	Val	GUC	149	0.4968
Gly	GGU	486	1.2656	Pro	CCU	336	1.4852	Val	GUG	183	0.61
His	CAC	133	0.5362	Gln	CAA	597	1.5984	Val	GUU	454	1.5132
His	CAU	363	1.4638	Gln	CAG	150	0.4016	Trp	UGG	381	1
								Tyr	UAC	129	0.3458
								Tyr	UAU	617	1.6542

## Data Availability

All original data (including sequencing reads and annotated genomes) supporting the reported results have been submitted to NCBI GenBank (Submission ID: 2997594). The corresponding GenBank accession numbers will be provided once they are assigned, and the data can be accessed via the NCBI platform.
